# Accurate noise-robust classification of Bacillus species from MALDI-TOF MS spectra using a denoising autoencoder

**DOI:** 10.1515/jib-2023-0017

**Published:** 2023-11-20

**Authors:** Yulia E. Uvarova, Pavel S. Demenkov, Irina N. Kuzmicheva, Artur S. Venzel, Elena L. Mischenko, Timofey V. Ivanisenko, Vadim M. Efimov, Svetlana V. Bannikova, Asya R. Vasilieva, Vladimir A. Ivanisenko, Sergey E. Peltek

**Affiliations:** Federal Research Center Institute of Cytology and Genetics SB RAS, 630090 Novosibirsk, Russia; Kurchatov Center for Genome Research, Institute of Cytology and Genetics SB RAS, 630090 Novosibirsk, Russia; Novosibirsk State University, 630090 Novosibirsk, Russia

**Keywords:** classification of Bacillus species, MALDI-TOF, denoising autoencoder, random forest

## Abstract

Bacillus strains are ubiquitous in the environment and are widely used in the microbiological industry as valuable enzyme sources, as well as in agriculture to stimulate plant growth. The Bacillus genus comprises several closely related groups of species. The rapid classification of these remains challenging using existing methods. Techniques based on MALDI-TOF MS data analysis hold significant promise for fast and precise microbial strains classification at both the genus and species levels. In previous work, we proposed a geometric approach to Bacillus strain classification based on mass spectra analysis via the centroid method (CM). One limitation of such methods is the noise in MS spectra. In this study, we used a denoising autoencoder (DAE) to improve bacteria classification accuracy under noisy MS spectra conditions. We employed a denoising autoencoder approach to convert noisy MS spectra into latent variables representing molecular patterns in the original MS data, and the Random Forest method to classify bacterial strains by latent variables. Comparison of the DAE-RF with the CM method using the artificially noisy test samples showed that DAE-RF offers higher noise robustness. Hence, the DAE-RF method could be utilized for noise-robust, fast, and neat classification of Bacillus species according to MALDI-TOF MS data.

## 1 Introduction

Representatives of the Bacillus genus comprise Gram-positive aerobic or facultative anaerobic rod-shaped bacteria, ubiquitous in the environment (soil, air, and water) [1]. They serve as widespread sources of industrial enzymes for the food, textile, and chemical industries [2]. They are also used as hosts for recombinant gene expression [3] and as a source of recombinant genes [4]. Bacillus strains show promise for agricultural use as rhizobacteria stimulating plant growth [5] and find application in disinfection systems [6, 7].

The Bacillus genus encompasses several closely related groups of species. The intragroup similarity between which can exceed 99 % for 16S rRNA, as seen in *Bacillus subtilis* [8]. Notably, the *Bacillus cereus* group comprises *Bacillus cereus*, *Bacillus anthracis*, and *Bacillus thuringiensis*, which are genetically very similar yet considered separate species due to differing pathogenicity [9]. *Bacillus safensis*, a new species, was isolated from *Bacillus pumilus* based on the gyrB gene sequence [10]. A polyphasic taxonomy approach led to the description of three additional species: *Bacillus altitudinis*, Bacillus stratosphaericus, and *Bacillus aerophilus* [11]. These species exhibit closely related 16S rRNA gene sequences, forming the *B. pumilus* group [12]. Existing approaches make rapid classification of such species challenging and an urgent task. Traditional methods for microorganism identification, such as biochemical tests and DNA sequencing, are time-consuming and labor-intensive. A breakthrough in identifying a broad spectrum of bacterial species has emerged through the application of matrix-assisted laser desorption-ionization time-of-flight mass spectrometry (MALDI-TOF MS) [13, 14]. Currently, MALDI-TOF MS is increasingly utilized in clinical laboratories for the pathogenic strains identification and for characterizing of environmental and food microbiota [15–22]. By generating mass spectra that quantify proteins and peptides in a pure microorganism culture, MALDI-TOF MS creates species-specific fingerprints, enabling accurate strain identification at genus and species levels [23, 24].

MALDI-TOF MS has successfully characterized and profiled the Bacillus genus, including *Bacillus cereus*, *Bacillus licheniformis*, and *Bacillus subtilis* [25, 26], and it has discriminated between members of the *Bacillus cereus* group [27–29]. Additionally, it has distinguished closely related species of biotechnological and pharmaceutical importance, such as Bacillus pumilis and *Bacillus safensis*, which are traditionally challenging to separate [30].

Recently, machine learning (ML)-based methods have increasingly been used to deal with bacterial strains identification problems [31]. For instance, Desaire et al. [32] developed an ML method for classifying mass spectrometry (MS) data from glycomics experiments using the Aristotle Classifier. Roux-Dalvai et al. [33] proposed a method for identifying bacterial strains in the urine based on LC-MS/MS peptide signature data, employing ML classifiers such as NaiveBayes, BayesNet, and Hoeffding tree. In a case study, the XGBoost classifier played a crucial role in identifying polymicrobial species based on MS data regarding their membrane glycolipids [34]. To enhance the characterization of very similar bacteria spectra, support vector machines, random forest classifiers, and new resampling methods have been introduced [35]. In a large-scale comparative study conducted by Mortier et al. [36], bacterial identification using MALDI-TOF mass spectrometry and ML methods, including univariate convolutional neural networks, hierarchical classifiers, and out-of-distribution detection was explored. The authors suggested the use of Monte Carlo dropout neural networks for bacterial identification, which have proven successful in other areas such as computer vision. Applying traditional ML algorithms to analyze MALDI-TOF MS data often necessitates addressing a dimensionality reduction problem. Dimensionality reduction becomes especially important when training ML models with a dataset characterized by a relatively small sample size. It is known that with small training samples, due to the high dimensionality of the MS data, the detection model is subject to overfitting [37].

Data compression aims to convert data into a reduced yet quality-preserving representation, facilitating the capturing and visualization of underlying latent variables. In particular, these latent variables uncover molecular patterns. Reflecting clusters of similar spectra with potential biological significance [38].

Numerous methods for data dimensionality reduction, specific to certain subject areas, have been developed. At the same time, many traditional methods such as Principal Component Analysis (PCA), Non-Negative Matrix Factorization (NNMF), and Latent Dirichlet Distribution (LDA) come with limitations tied to their linearity. Nonlinear dimensionality reduction methods such as t-distributed stochastic neighbor embedding (t-SNE) have gained popularity in recent years for omics data analysis [39–42]. Nevertheless, these methods fall short in projecting new data into an already computed embedding. Neural network-based autoencoder methods have shown promise for efficient non-linear dimensionality reduction, thus fitting well into deep learning approaches [43, 44]. Several autoencoder architecture variants have been developed, including convolutional, regularized, variational, sparse, multilevel, deep, and generative, among others [43]. Variational autoencoders, which represent a probabilistic generative model learning an unsupervised and non-linear parametric mapping between high and low dimensional spaces, have been effectively applied to the analysis of omics data, including single-cell data [45], and medical image segmentation [46].

Utilizing autoencoders for the analysis of mass spectrometric data is a promising approach. Specifically, a fully connected variational autoencoder neural network has been employed for the analysis and peak learning of mass spectrometric imaging (MSI) data [38]. Based on this neural network model, the authors developed the msiPL deep learning tool. Li et al. [47] applied a denoising autoencoder to accurately classify Listeria species using MALDI-TOF mass spectrometry. In an earlier study, we proposed a centroid method (CM) to mass spectrometry data processing that represented the mass spectrum as a vector in multidimensional Euclidean space, using the Jaccard index [48, 49]. We applied the proposed method to identify microorganisms by analyzing 24 strains belonging to the *B. pumilus* group. This approach enabled us to confidently divide the strains into two groups corresponding to the closely related species, *Bacillus pumilus* and *Bacillus altitudinis*.

In this article, we have adopted a denoising autoencoder (DAE) approach to classify closely related microorganisms of the *Bacillus pumilus* group. The concept of denoising autoencoders involves training the DAE with generated noisy MS spectra as the input, from which the original MS spectra are predicted. It is anticipated that this method will enhance the classification method’s robustness to the variability of MS spectra in Bacillus strains.

We applied this approach to analyze the MALDI-TOF MS spectra of 19 species of the genus Bacillus. In addition, *E. coli* was included in the analysis. All spectra were sourced from Starostin [49]. Microorganism classification was conducted based on the latent space coordinates presented in the hidden DAE layer, using Random Forest (RF). To assess the level of noise introduced into the original spectra during DAE training, we analyzed the observed intraspecific variability of the spectra in the examined samples. The analysis revealed a variability of the spectra ranging from 0.1*C*_*o*_ to 1.0*C*_*o*_, where *C*_*o*_ represents the peak size. DAE was trained using original data with zero mean normal noise and a variance of 0.4*C*_*o*_. To test the resulting models, we generated eleven independent random samples with noise levels ranging from 0.1*C*_*o*_ to 2.0*C*_*o*_ . In comparison with the previously developed CM, the DAE-RF method demonstrated a greater robustness to noise in mass spectra. The maximum classification accuracy (F1) for the DAE-RF was 0.99, whereas for the CM model, it was 0.89.

## 2 Materials and methods

### 2.1 MALDI-TOF MS spectra

The MALDI-TOF MS spectra, used for analysis, were taken from Starostin [49]. A total of 152 spectra were obtained for 70 strains representing 19 species of the Bacillus genus (Table 1). In addition to the Bacillus strains, an *E. coli* strain was included.

**Table 1:** MALDI-TOF MS data used in the analysis.

No	Species	Number of strains	Number of MALDI-TOF MS spectra
1.	*Bacillus pumilus*	18	35
2.	*Bacillus altitudinis*	9	19
3.	*Bacillus licheniformis*	8	21
4.	*Bacillus cereus*	8	16
5.	*Bacillus megaterium*	6	12
6.	*Bacillus flexus*	3	5
7.	*Bacillus thuringiensis*	2	4
8.	*Geobacillus subterraneus*	2	4
9.	*Bacillus atrophaeus*	2	4
10.	*Bacillus simplex*	2	12
11.	*Bacillus weihenstephanensis*	1	2
12.	*Bacillus aryabhattai*	1	2
13.	*Bacillus berkeleyi*	1	2
14.	*Bacillus subtilis*	1	2
15.	*Bacillus mycoides*	1	2
16.	*Bacillus coagulans*	1	2
17.	*Bacillus clausii*	1	2
18.	*Anoxybacillus flavithermus*	1	2
19.	Bacillus chungangenis	1	2
20.	*E. coli*	1	2
	Total	152	71

### 2.2 Generation of noisy MALDI-TOF MS data test samples

Noisy spectra were generated by adding a random number from the normal distribution to each component of the spectrum according to the following formula:

$$C_{n}=C_{o}+e,$$

where *C*_*n*_ represents the noisy peak, *C*_*o*_ is the original peak, *e* belongs to a normal distribution with parameters *N* (*a* = 0, σ = *d·C*_*o*_), *d* being the noise level factor.

Additionally, we established boundary conditions for *C*_*n*_ values. For negative values of *C*_*n*_, the modulus was used. The upper threshold for positive values of *C*_*n*_ was set at 1.

### 2.3 Estimation of intraspecies variability of mass spectra

The range of values for the noise level factor *d* was estimated by analyzing the intraspecies variability of MS spectra using the following formula:

$$d_{\text{aver}}\left(q\right)=\frac{1}{n_{q}}\sum \frac{\frac{1}{m_{q}}\sum {\left(Lo_{ij}-Lav_{j}\right)}^{2}}{Lav_{j}},$$

where *q* ∈ [1, *k*], *k* is the number of species with more than 4 spectra presented,

*i* ∈ [1, *m*_*q*_], *m*_*q*_ is the number of spectra for the *q*th species,

*j* ∈ [1, *n*_*q*_], *n* is the number of spectrum components with nonzero *Lav*_*j*_,

*Lo*_*i,j*_ is the value of the *j*th component of the *i*th spectrum,

*Lav*_*j*_ is the average value of *Lo*_*i,j*_ over all spectra *j*.

Species with at least five mass spectra were analyzed (Table 1). The resulting range of *d* values was used to add noise to the original mass spectra when training DAE models and to form test samples of mass spectra.

### 2.4 Denoising autoencoder (DAE)

Autoencoders are self-supervised neural network architectures used to perform data compression, taking into account an encoding, a decoding, and a distance functions [43]. The manifold learning performance of autoencoders can be significantly enhanced by augmenting the reconstruction loss using a regularization term [50–54].

To increase the autoencoder’s robustness to changes in input data, a special type of denoising autoencoder was proposed [55, 56]. The input to the DAE is data with added noise. The DAE encodes the input data and attempts to predict the original data before the noise was added.

In this work, we used the PyTorch library (https://pytorch.org) to create the DAE. The architecture of the encoder consisted of an input layer with a dimension of 12,001 nodes, two hidden layers with dimensions of which are 6000 and 750 nodes, and a final layer for latent space coordinates with a dimension of 50 feature points. The decoder’s architecture was symmetrical, with layer dimensions set in reverse order.

The rectified linear unit (ReLU) function was used as the activation function for the network layers. The mean-square error function served as the loss function. Adam was chosen as the optimizer [57], with parameters were set as standard (learning rate = 3e-4, parameter for the first exponential moving average = 0.9, for the second = 0.99). During DAE training, noisy MS spectra were generated using 151 original spectra as described above. We conducted 100 training epochs, with each batch being noisy. The batch size was set to 16. The training sample size was 70 % of the full dataset, with the remaining 30 % serving as the control sample.

In each training iteration, the DAE calculates the loss between the reconstructed noisy MS spectrum received from the decoder and the original noise-free MS spectrum, attempting to minimize the loss. The noise addition operation is applied only during training and not during prediction.

### 2.5 MS spectra classification with random forest

The DAE autoencoder was utilized to learn core features from the MALDI-TOF MS data. Following that, the Random Forest (RF) algorithm, using the scikit-learn [58], was applied to classify the MALDI-TOF MS data based on the extracted features. RF models used the encoded features with a length of 50 as input. MS spectra were classified into 20 classes corresponding to different Bacillus species. We used the RandomForestClassifier from the sklearn library with a default set of parameters (number of trees = 100, division criterion – Gini, minimum number of elements in a leaf for division = 2). The bootstrap method was used to assess the accuracy of the regression models [59]. The encoded features were divided into a training set and a test set in a 70:30 ratio, respectively.

The importance of features was calculated using the feature_importance_procedure of the sklearn library. The Gini importance (mean decrease impurity) was estimated from the Random Forest structure.

### 2.6 Estimation of the classification accuracy

The F1 score was used to assess the accuracy of classification, calculated using the following formulas:

$$\text{Recall}=\text{TP}/\left(\text{TP}+\text{FN}\right),$$

$$\text{Precision}=\text{TP}/\left(\text{TP}+\text{FP}\right),$$

$$\text{F}1=2*\text{Precision*}\text{Recall}/\left(\text{Precision }+\text{ Recall}\right),$$

where TP represents true positives, FP false positives, TN true negatives, and FN false negatives.

## 3 Results

**Figure 1:**
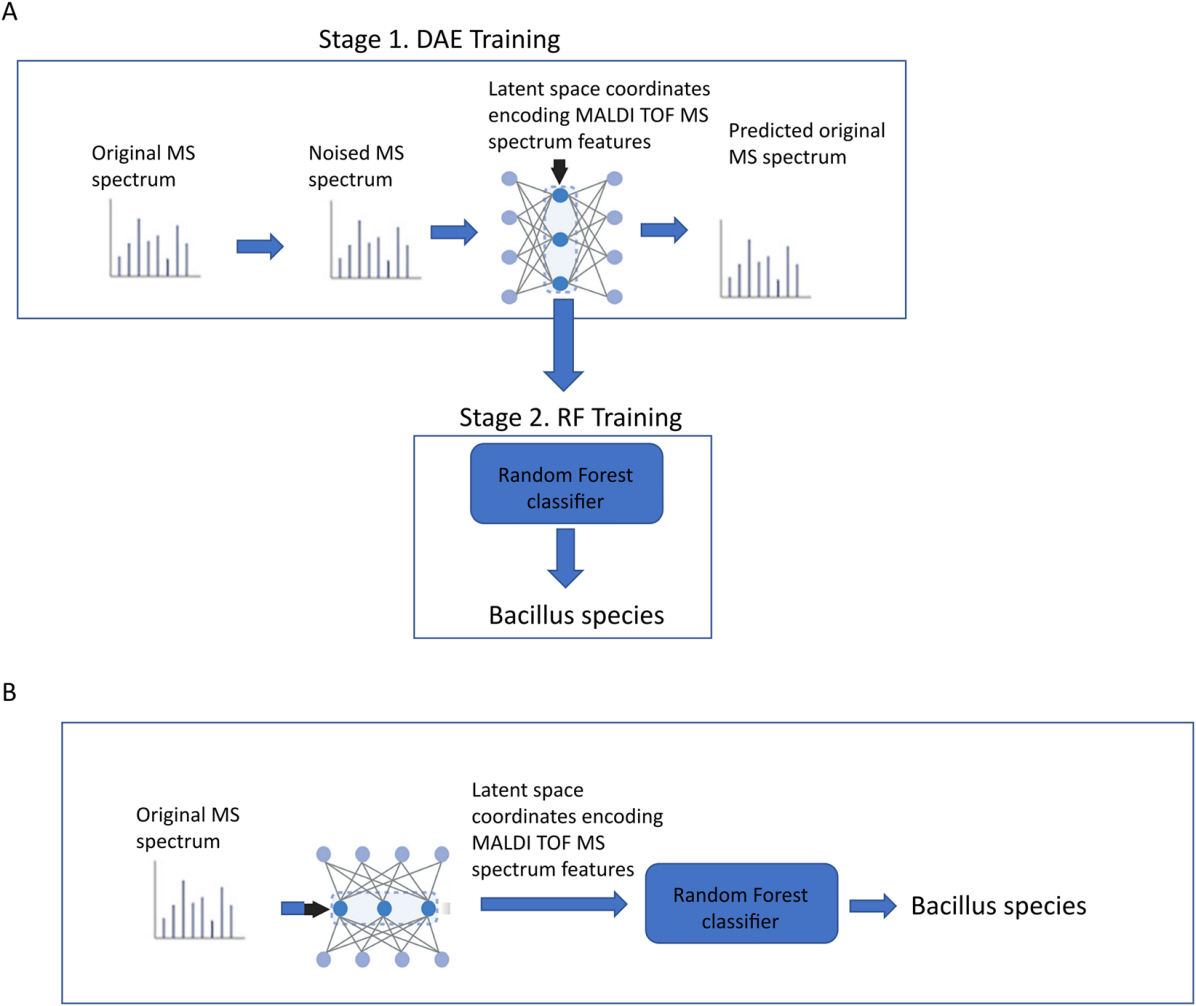
The used workflow of the microorganism classification approach, indicating the training stage of DAE and RF models (A), as well as the application of the trained models for the classification of Bacillus species according to MS spectra (B).

The classification of MALDI-TOF MS spectra was carried out using mass spectrometric analysis data from 20 species, including 70 Bacillus strains and an *E. coli* strain, published by us earlier [49]. Each strain was represented by two or more replicates. The total MALDI-TOF MS data consisted of 152 mass spectra (Table 1). The classification of microorganisms was performed by successively applying the Denoising Autoencoder (DAE) and Random Forest (RF) models (Figure 1).

In the first stage, the DAE was used to learn core features from the MALDI-TOF MS data. In the second stage, the RF models were applied to classify the MALDI-TOF MS data. The RF models took the DAE-encoded features of length 50 as input. By convention, the class with the highest probability was deemed the predicted class.

### 3.1 DAE training

DAE training was carried out as depicted in Figure 1A. The original MS spectra were subjected to noise and then fed into the neural network. The noise was introduced by adding a random number from the normal distribution to each component of the original spectrum (see formula 1). The variability of the mass spectra within individual Bacillus species was assessed by calculating the *d* index according to formula 2. An analysis of the intraspecies variability of the mass spectra revealed that the noise parameter *d* varies within the range from 0.1 to 1.0. For further work, we selected the value *d* = 0.4, which corresponds to approximately half of the range calculated within the species variability. This value was chosen to keep the noisy spectra within the limits of natural variability.

Noisy MS spectra were fed into the DAE. The loss function was calculated based on the differences between the predicted spectrum and the original noise-free MS spectrum. When utilizing trained DAE models to classify MS spectra from test samples, the input spectra were not subject to noise (Figure 1B).

**Figure 2:**
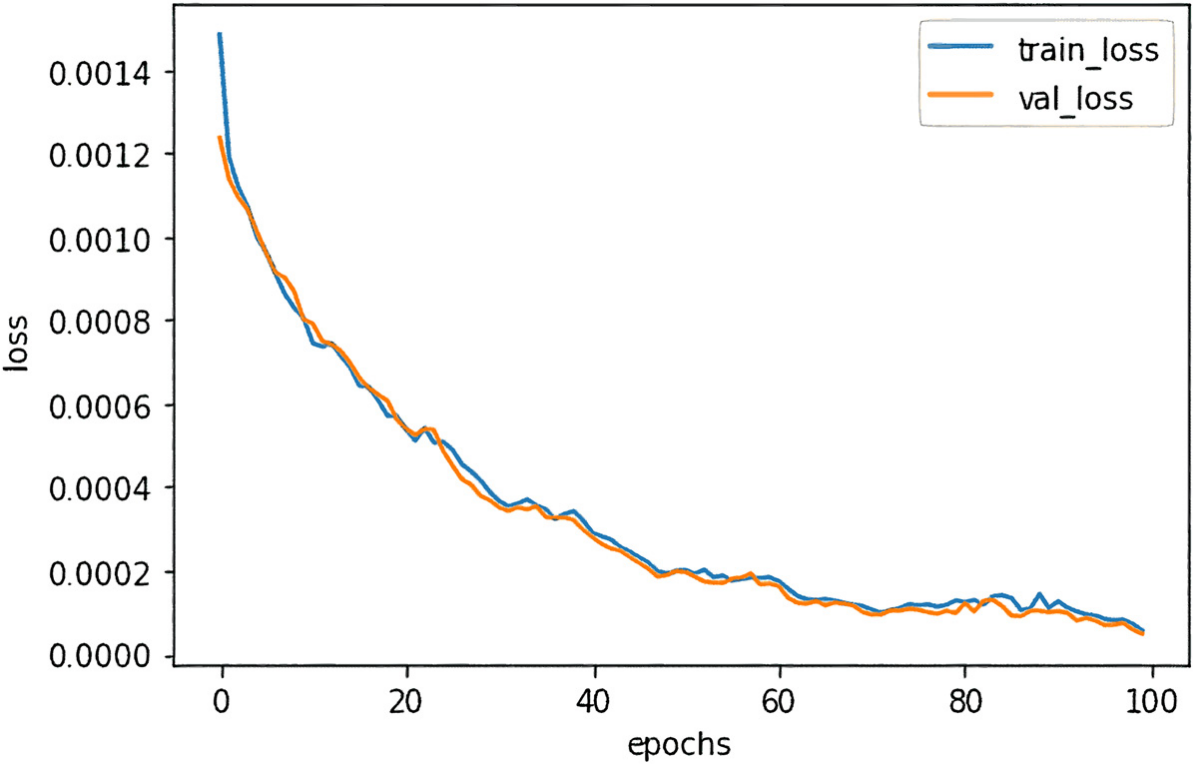
DAE training loss function plots for noise level *d* = 0.4.

The dynamics of the loss function during DAE training at a noise level of *d* = 0.4 is shown in Figure 2. The figure indicates a tendency for the values of the loss function to decrease with an increase in the number of training epochs. A similar trend was observed for other noise levels.

**Figure 3:**
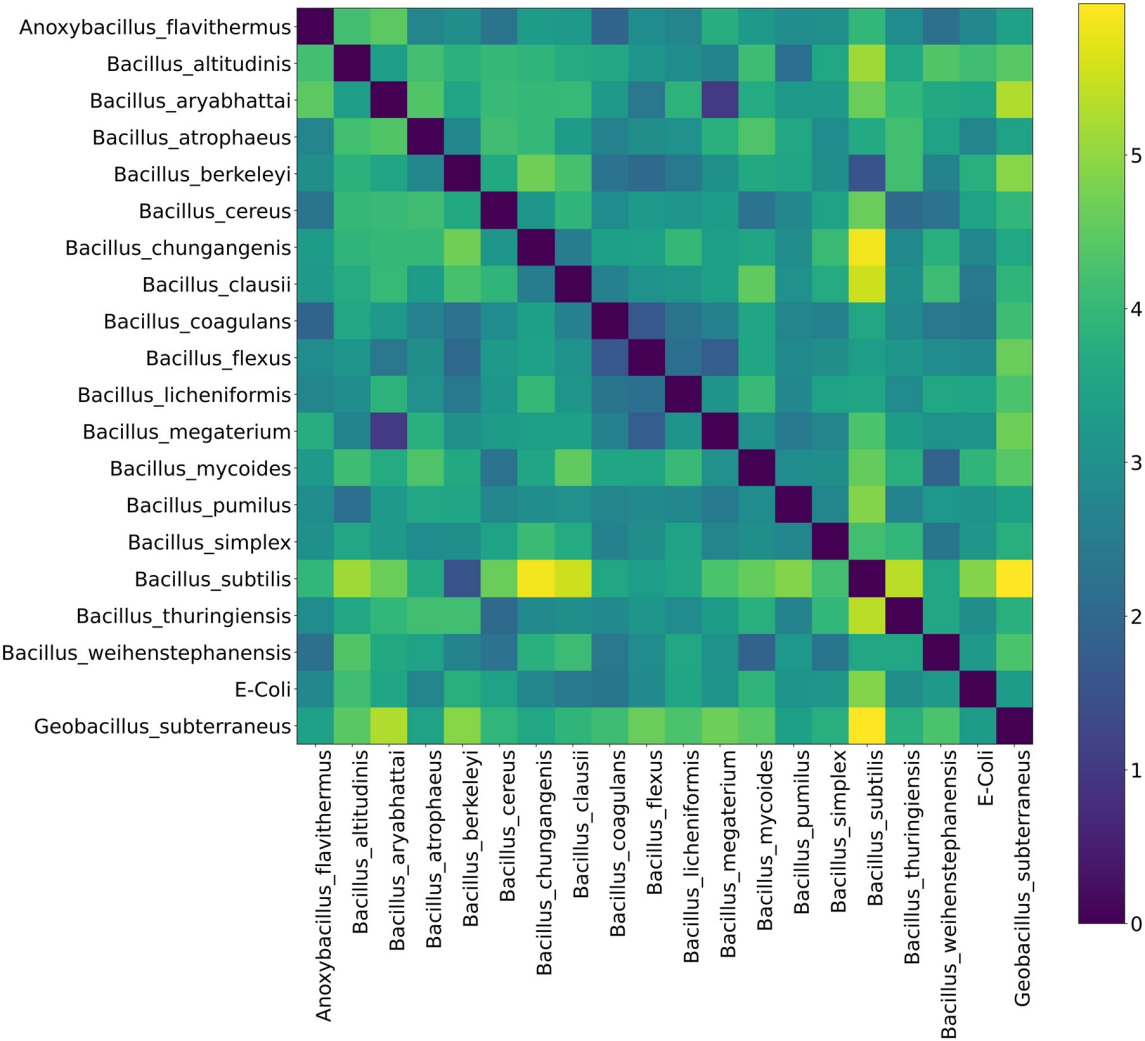
Heatmap representation of intra- and inter-species distances of the analyzed microorganisms according to the latent space coordinates of the DAE model. Average intra-species distances are represented by diagonal elements, and inter-species distances are represented by off-diagonal elements.

Figure 3 shows the potential ability of the encoded DAE model features to classify the microorganisms under consideration. The figure indicates that intra-species distances are characterized by smaller values compared to inter-species ones. This suggests that the latent variables, which capture molecular patterns in MS data, are significant for the classification of Bacillus species.

### 3.2 Classification of mass spectra using random forest

**Figure 4:**
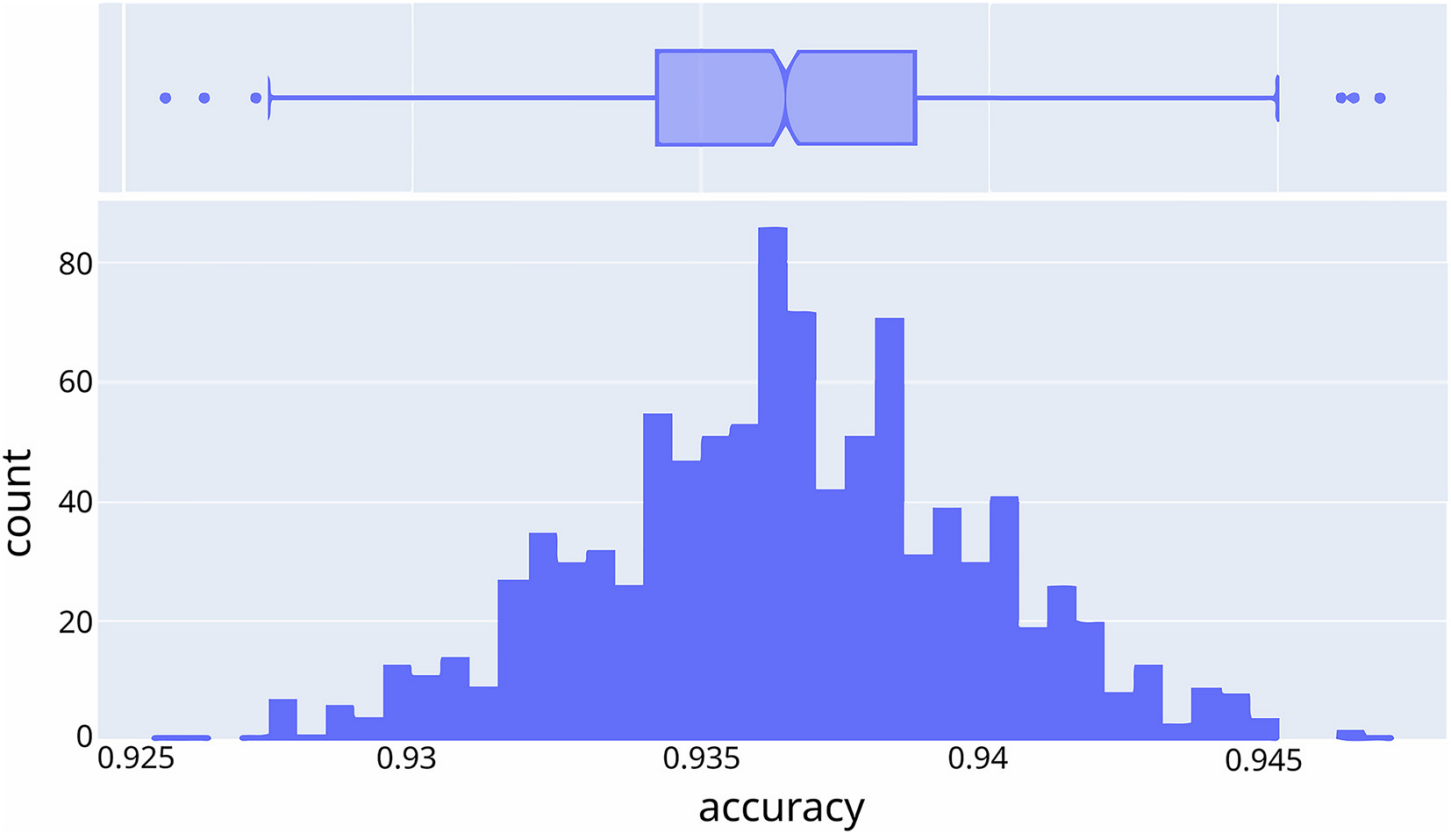
Assessment of classification accuracy by the RF model using the bootstrap method.

The RF input was the latent space coordinates provided by DAE. The distribution of average classification accuracy of the analyzed microorganisms by Bacillus species, calculated using the bootstrap method during RF model training, is shown in Figure 4.

A total of 1000 accuracy estimates were made on test sets, each accounting for 30 % of all spectra used in RF training. The median of the distribution corresponds to 93.8 % accuracy. The values of different classification accuracy indicators for each Bacillus species, obtained using the trained RF model on the entire volume of the training sample, are presented in Table 2.

**Table 2:** Accuracy of classification of microorganisms by species.

Species	Precision	Recall	F1-score
*Anoxybacillus flavithermus*	1.000	1.000	1.000
*Bacillus altitudinis*	0.877	0.777	0.824
*Bacillus aryabhattai*	0.567	0.930	0.705
*Bacillus atrophaeus*	1.000	1.000	1.000
*Bacillus berkeleyi*	0.917	0.967	0.934
*Bacillus cereus*	0.900	0.899	0.899
*Bacillus chungangenis*	1.000	1.000	1.000
*Bacillus clausii*	1.000	1.000	1.000
*Bacillus coagulans*	1.000	1.000	1.000
*Bacillus flexus*	1.000	1.000	1.000
*Bacillus licheniformis*	0.800	1.000	0.889
*Bacillus megaterium*	0.987	0.882	0.931
*Bacillus mycoides*	0.990	1.000	0.995
*Bacillus pumilus*	0.943	0.927	0.935
*Bacillus simplex*	0.946	1.000	0.972
*Bacillus subtilis*	0.500	1.000	0.667
*Bacillus thuringiensis*	0.917	0.967	0.934
*Bacillus weihenstephanensis*	1.000	1.000	1.000
*E-Coli*	1.000	1.000	1.000
*Geobacillus subterraneus*	1.000	1.000	1.000

The table shows that the model effectively separates the samples by genus (*E-Coli*, *Geobacillus, Anoxybacillus* have an f1-score of 1.0). Interspecific differences within the *Bacillus* genus have an f1-score ranging from 0.667 to 1.0.

## 4 Discussion

In earlier work [48, 49], we proposed the use of the centroid method (CM) within a geometric framework based on linear transformation of the feature space to classify bacterial mass spectrometric analysis spectra. The CM method demonstrated good discrimination between two closely related Bacillus species (*B. pumilus* and *B. altitudinis*), which share over 98 % homology in the 16s rRNA gene sequence [48, 49]. In this study, a denoising autoencoder was used to tackle the problem of bacterial classification under noisy MS spectra. The first step used the DAE workflow to convert noisy MS spectra into latent variables representing the molecular patterns in the original MS data (Figure 1A). In the second step, the encoded MS spectra features were used to classify bacterial strains using Random Forest (Figure 1B).

To compare DAE-RF with CM, we applied both approaches to test sets of artificially noisy MALDI TOF MS spectra. The original MS data, containing 152 spectra of 70 *Bacillus* strains and an *E. coli* strain, were taken from [49]. Based on these, we formed 11 test sets of noisy mass spectra with different noise levels, specified by the parameter *d* in the range from 0.1 to 2.0.

The results on the classification accuracy of Bacillus strains using DAE-RF and CM methods are shown in Table 3. DAE-RF significantly outperformed the CM method. At noise levels within observed within-species variability (*d* ∈ [0.1, 1.0]), DAE-RF maintained high accuracy, while CM showed a sharp drop in F1 values with increasing noise in the test spectra. Even at noise levels exceeding 1.0, DAE-RF, despite a steeper decline in accuracy compared to the range of d values for observed within-species variability, was still markedly more accurate than the CM method. Thus, DAE-RF demonstrates higher classification robustness to noise in the original spectra compared to the previously proposed CM method.

**Table 3:** Classification accuracies of Bacillus species using DAE-RF and CM methods in terms of F1, depending on the level of noise of the original MS spectra.

Noise level (d)	DAE-RF	CM
0.1	0.99	0.89
0.15	0.99	0.81
0.2	0.93	0.62
0.25	0.93	0.51
0.3	0.86	0.45
0.4	0.84	0.21
0.6	0.80	0.17
0.8	0.80	0.13
1.0	0.73	0.09
1.5	0.59	0.08
2.0	0.55	0.056

## 5 Conclusions

The use of machine learning methods, including dimensionality reduction in MALDI TOF MS data with noise suppression using denoising autoencoders, in the first step, and spectrum classification using Random Forest, in the second step, facilitated the accurate classification of Bacillus species from noisy test samples. DAE-RF can be used for robust Bacillus strain classification, even in the face of mass spectrum variability caused by changing conditions during MS spectrum measurements, as well as natural within-species spectrum variability. Future plans include integrating the developed DAE-RF method into the Online Platform for Identification of Microorganisms software system (http://biotyper.sysbio.ru).
